# Intestinal invasion and disseminated disease associated with *Penicillium chrysogenum*

**DOI:** 10.1186/1476-0711-4-21

**Published:** 2005-12-21

**Authors:** Adrian L Barcus, Steven D Burdette, Thomas E Herchline

**Affiliations:** 1 Department of Medicine, Wright State University School of Medicine, Dayton, Ohio, USA; 2 Division of Infectious Diseases, Department of Medicine, Wright State University School of Medicine, Dayton, Ohio, USA

## Abstract

**Background:**

*Penicillium *sp., other than *P. marneffei*, is an unusual cause of invasive disease. These organisms are often identified in immunosuppressed patients, either due to human immunodeficiency virus or from immunosuppressant medications post-transplantation. They are a rarely identified cause of infection in immunocompetent hosts.

**Case presentation:**

A 51 year old African-American female presented with an acute abdomen and underwent an exploratory laparotomy which revealed an incarcerated peristomal hernia. Her postoperative course was complicated by severe sepsis syndrome with respiratory failure, hypotension, leukocytosis, and DIC. On postoperative day 9 she was found to have an anastamotic breakdown. Pathology from the second surgery showed transmural ischemic necrosis with angioinvasion of a fungal organism. Fungal blood cultures were positive for Penicillium chrysogenum and the patient completed a 6 week course of amphotericin B lipid complex, followed by an extended course oral intraconazole. She was discharged to a nursing home without evidence of recurrent infection.

**Discussion:**

*Penicillium *chrysogenum is a rare cause of infection in immunocompetent patients. Diagnosis can be difficult, but *Penicillium *sp. grows rapidly on routine fungal cultures. Prognosis remains very poor, but aggressive treatment is essential, including surgical debridement and the removal of foci of infection along with the use of amphotericin B. The clinical utility of newer antifungal agents remains to be determined.

## Case report

A 51 year old African-American female, eight years status post colostomy secondary to diverticulitis, presented to the emergency department with a 48 hour complaint of abdominal pain, nausea and vomiting. Vitals signs were initially stable, physical exam was significant for distended, tender abdomen. A CT scan revealed extraluminal intraperitoneal air and the patient was taken to the operating room. Exploratory laparotomy revealed an incarcerated peristomal hernia with perforated small bowel. Extensive enterolysis, small bowel resection and hernia repair with permacol were performed. Cultures grew *Escherichia coli*; pathology revealed only neutrophilic inflammation. Due to the severity of disease, and distention of small bowel, the abdomen was left open to heal by secondary intention. Her postoperative course was complicated by severe sepsis syndrome with respiratory failure, hypotension, leukocytosis, and DIC. Broad spectrum antibiotics were administered. The patient was also treated with hydrocortisone 100 mg every 8 hours and subsequently developed steroid induced hyperglycemia. On postoperative day (POD) 3, a vacuum assisted closure (VAC) device was placed to assist with fascial healing. The sepsis syndrome resolved and the patient's condition improved until POD 9, when stool was noticed collecting within the VAC system. The patient was returned to the operating room where she was found to have an anastamotic breakdown.

**Figure 1:**
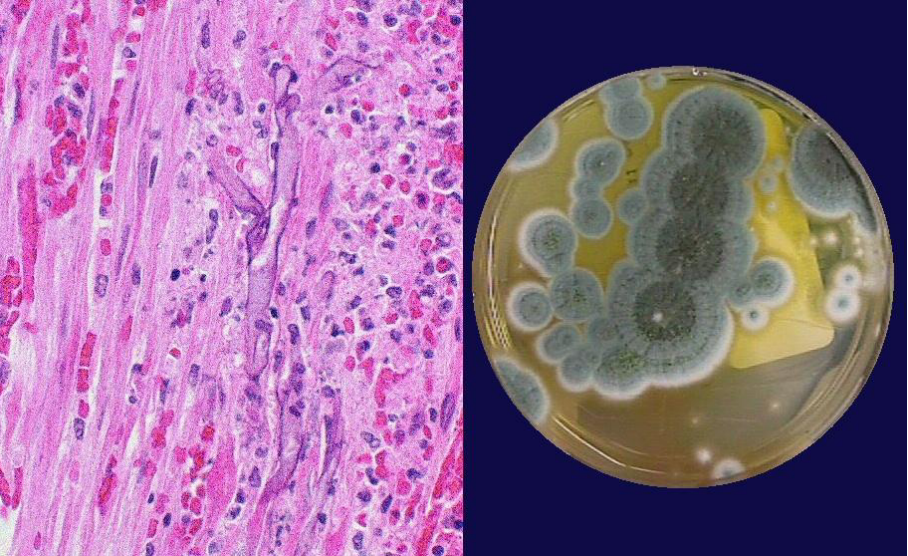
**Left panel**: H&E stain of small bowel mucosa showing angioinvasion. **Right panel**: Characteristic appearance of *Penicillium *on culture media.

Pathology from the second surgery showed transmural ischemic necrosis with angioinvasion of a fungal organism (see figure 1). Fungal blood cultures were obtained and the patient was started on amphotericin B lipid complex. Within 48 hours, fungal blood cultures were positive for *Penicillium *sp. The patient completed a 6 week course of amphotericin B lipid complex, followed by an extended course oral intraconazole. The patient was eventually transferred to a long-term rehabilitation facility, without evidence of recurrent infection. The only identified risk factors for invasive Penicillium infection were stress dose steroids and broad spectrum antibiotics. Final fungal identification demonstrated the organism to be *Penicillium chrysogenum*^A^. In vitro susceptibility at 24 and 48 hrs respectively was reported as: amphotericin B 0.5 μg/ml and 1.0 μg/ml, itraconazole 0.06 μg/ml and 0.25 μg/ml, and voriconazole 0.25 μg/ml and 1 μg/ml^B^.

## Discussion

*P. marneffei *is a common cause of infection in the HIV population in endemic areas of Southeast Asia. Infections caused by other *Penicillium *species are rare [1]. A recent review found only 34 cases. Sources of infection included peritonitis in patients on peritoneal dialysis, endocarditis, endophthalmitis, fungemia, esophagitis, and pulmonary infection [2].

The genus *Penicillium *comprises approximately 225 species, including *P. chrysogenum (also called P. notatum)*, the source of penicillin, and other species used in making cheeses. They are a widespread group, found in soil, decaying vegetation and in air. On microscopic examination *Penicillium *has hyaline and septated hyphae (in contrast with *Mucor *which is non-septated) [3,4]. Their appearance on culture media is pictured in the figure, with characteristic green-blue color, velvety appearance, and radial grooves at 25°C [4,5].

In general, *Penicillium *species have low pathogenicity and infection is usually seen in immunocompromised individuals. Isolated cases have been reported without clear immune suppression. The clinical presentation of disease caused by *Penicillium *is diverse, and it is known to cause pathology via direct infection, hypersensitivity reaction [6], and pulmonary fibrosis [7]. In the case of infection, the breadth of presentation is illustrated in the case review by Lyratzopoulos et al. [2], which found cases of pulmonary infection including lobar pneumonia, bronchiolitis obliterans organizing pneumonia, localized granuloma, fungus ball, and diffuse parenchymal infection. Cardiac infection was primarily related to instrumentation or procedures (most commonly valve surgery with subsequent endocarditis) [3,8]. Intra-abdominal infection was predominantly associated with peritoneal dialysis [9,10]. The remaining infections included intracranial infection, endophthalmitis, cutaneous infection, and infectious esophagitis. This distribution of infection suggests that the organism can disseminate through hematogenous as well as direct mucosal invasion.

The primary difficulty in diagnosing infections by *Penicillium*, due to its rarity, is a low clinical suspicion. It should be considered in the situation of immune compromise or after appropriate antibiotic course without resolution of clinical symptoms. There are no clear indicators from history or physical exam that are specific for *Penicillium *infection, and it can cause a wide spectrum of disease. Fortunately the organism often grows rapidly from normal fungal cultures [4], and thus it may be identified in the course of a normal workup. Results from a biopsy, or as in this case, surgical pathology, may be helpful when available. However, one must also consider that positive cultures from respiratory sources may not be associated with invasive disease, but rather colonization [2]. Case reports do not describe any specific imaging modalities that aid in diagnosis [2], but imaging may play a role in localization of foci of infection.

Multiple antifungal agents have been used with various lengths of treatment for *Penicillium *infection. Amphotericin B and itraconazole has been used with mixed results, no doubt in part due to the variety of sites of infection, and co-morbidity/underlying illnesses [2]. Combination therapies with the aforementioned antifungal agents (including ketoconazole) or flucytosine have also been used with variable results. In vitro data suggests that newer agents, such as Echinocandins (such as caspofungin or micafungin), triazoles (such as voriconazole) and terbinafine have activity, though clinical data is still lacking [11,12]. At this time the best evidence appears to support amphotericin B in conjunction with strategies aimed at removing the focus of infection, such as removal of lines, replacement of infected valves, or surgical debridement of affected tissue. There is no standard duration of therapy (reports range from 2–12 weeks of treatment) [2] and each case needs to be individualized to determine appropriate length of antifungal administration. Even with these interventions, the prognosis for *Penicillium *infections remains poor.

This case highlights *Penicillium *as one of many causes of infection in immunocompromised patients. Diagnosis can be difficult, but *Penicillium *grows rapidly on routine fungal cultures. Prognosis remains very poor, but aggressive treatment is essential, including surgical debridement and the removal of foci of infection along with the use of amphotericin B. The clinical utility of newer antifungal agents remains to be determined.

## Footnote

A. Identification of Penicillium chrysogenum confirmed by Maren Klich, USDA, Southern Regional Research Center, New Orleans, LA.

B. Sensitivity testing provided by Fungus Testing Laboratory, San Antonia, Texas.

## Competing interests

The author(s) declare that they have no competing interests.

## Authors' contributions

ALB: Primary author of the manuscript.

SDB: Examined and treated the affected patient, critical appraisal and editing of manuscript.

TEH: Examined and treated the affected patient, critical appraisal and editing of manuscript.

All authors approved of final manuscript submission.

## Financial support

None.
